# Functional switching of ascorbate peroxidase 2 of rice (OsAPX2) between peroxidase and molecular chaperone

**DOI:** 10.1038/s41598-018-27459-1

**Published:** 2018-06-15

**Authors:** Sung Hyun Hong, Bhumi Nath Tripathi, Moon-Soo Chung, Chuloh Cho, Sungbeom Lee, Jin-Hong Kim, Hyoung-Woo Bai, Hyeun-Jong Bae, Jae-Young Cho, Byung Yeoup Chung, Seung Sik Lee

**Affiliations:** 1 Advanced Radiation Technology Institute, Korea Atomic Energy Research Institute, 29 Geumgu-gil, Jeongeup, 56212 Korea; 2 Department of Bioenergy Science and Technology, Chonnam National University, Gwangju, 61186 Korea; 3 Department of Bioenvironmental Chemistry, Chonbuk National University, 567 Baekje-daero, Deokjin-gu, Jeonju 54896 Korea; 4 Department of Radiation Biotechnology and Applied Radioisotope, Korea University of Science and Technology, Daejeon, 34113 Korea

## Abstract

Ascorbate peroxidase (APX) is a class I haem-containing peroxidase, which catalyses the conversion of H_2_O_2_ to H_2_O and O_2_ using ascorbate as the specific electron donor. APX plays a central role in the elimination of intracellular reactive oxygen species (ROS) and protects plants from the oxidative damage that can occur as a result of biotic and abiotic stresses. At present, the only known function of APX is as a peroxidase. However, in this study, we demonstrate that *Oryza sativa* APX2 also operates as a molecular chaperone in rice. The different functions of OsAPX2 correlate strongly with its structural conformation. The high-molecular-weight (HMW) complexes had chaperone activity, whereas the low-molecular-weight (LMW) forms displayed predominantly APX activity. The APX activity was effectively inhibited by sodium azide, which is an inhibitor of haem-containing enzymes, but this did not affect the protein’s activity as a chaperone. Additionally, the OsAPX2 conformational changes could be regulated by salt and heat stresses and these stimulated OsAPX2 dissociation and association, respectively. Our results provide new insight into the roles of APXs.

## Introduction

Rice is one of the most important crops in the world and is cultivated in a range of agro-climatic conditions. Therefore, rice plants encounter a variety of abiotic and biotic stresses. These stresses increase the production of reactive oxygen species (ROS) during adverse conditions and generate oxidative damage^1^. Most organisms, including rice, have evolved effective mechanisms for managing these potentially damaging increases in ROS. Ascorbate peroxidase (APX) is one of the most important of these defence components and can detoxify H_2_O_2._ Rice has eight *APX* genes and these encode enzymes that function in the cytosol (APX1 and APX2), peroxisome (APX3 and APX4), mitochondria (APX5 and APX6) and chloroplast (APX7 and APX8)^2–4^. Previous studies have demonstrated that a variety of APX enzymes can detoxify H_2_O_2_ in specific locations within different organisms in response to a range of external stimuli. The expression of these genes is regulated by a variety of stress factors, suggesting that APXs play important roles in plant stress resistance. Chloroplast APXs are generally more sensitive than cytosolic enzymes^4^ and although there are no corresponding data on rice APXs, chloroplastic APXs from *Arabidopsis thaliana* and green algae are highly susceptible to photooxidative stress^5,6^. Previous studies of mutations in cytosolic APX1 and APX2 confirmed that these enzymes are crucial for counteracting abiotic stress during plant growth and development^3,7^. Under normal growth conditions, *APX2* expression is difficult to detect but it is significantly upregulated in response to stress^8^. Overexpression of *APX2* is reportedly linked with improvements in salt tolerance in transgenic *Arabidopsis* and *Medicago sativa*^9,10^. Additionally, a gene knockout study demonstrated the critical role that OsAPX2 plays in rice plants growing under drought, high-salt or cold stress conditions^3^, and another study reported that transgenic alfalfa overexpressing the *OsAPX2* gene had an increased capacity for salt tolerance^11^.

We previously demonstrated, using an in-gel enzyme assay, that APX activity in the shoot tissue of a salt-sensitive rice cultivar (*Oryza sativa* L. cv. IR29) increased significantly in response to salt treatment^12^. During salt stress, the OsAPX1 and OsAPX2 isoenzymes played critical roles in eliminating H_2_O_2_ in the IR29 cultivar^13^. Our studies, using size exclusion chromatography (SEC) and native-polyacrylamide gel electrophoresis (PAGE), found that recombinant OsAPX1 and OsAPX2 proteins had a variety of forms with different molecular weights (data not shown). This resembled results from previous studies on 2-Cys peroxiredoxins (Prx) reported by König *et al*.^14^ and Jang *et al*.^15^. One of the Prx antioxidant proteins had a dual function as both a peroxidase and a chaperone and its enzymatic activity was regulated by its structural status^14–16^. The 2-Cys Prx regulated its own structure in a redox-dependent manner. The oxidized form of the 2-Cys Prx was a low-molecular-weight (LMW) dimer; however, reduced and hyper-oxidised dimers formed high-molecular-weight (HMW) complexes (e.g., decamers or dodecamers). These dynamic changes were closely related to functional switching between the protein acting as a peroxidase and a molecular chaperone. The LMW complexes had high peroxidase activity, whereas the HMW complexes functioned predominantly as chaperones.

To date, functional studies of APX proteins have been restricted to their antioxidant properties. However, in this study, we identify a novel function for APX proteins as molecular chaperones and also reveal the relationship between the structure and different functions of an APX protein. The different functions of OsAPX2 are strongly dependent on its structure. APX activity was greatest in the LMW protein forms, whereas the molecular chaperone activity predominated in the HMW complexes. Additionally, APX activity was not required for OsAPX2’s chaperone activity. The HMW complexes and LMW forms were regulated by heat stress *in vitro* and by salt treatment in the IR29 cultivar. Therefore, our results provide new insight into the functions of APXs to study in the future.

## Results

### OsAPX2 forms multiple structures of different sizes

**Figure 1:**
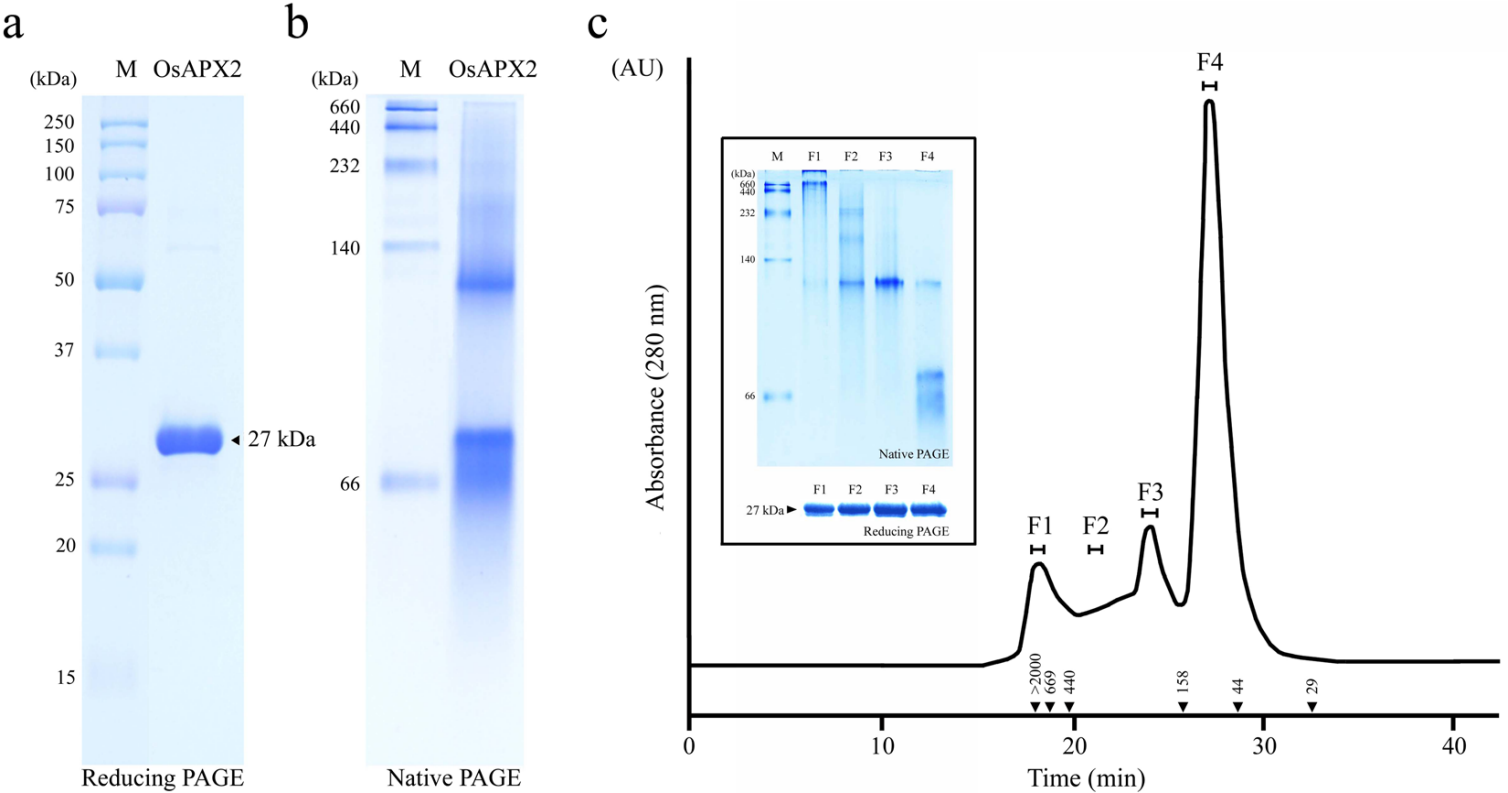
Structural analysis of the recombinant *Oryza sativa* ascorbate peroxidase 2 (OsAPX2) protein. The purified OsAPX2 protein was identified using 12% sodium dodecyl sulphate-polyacrylamide gel electrophoresis (SDS-PAGE) under reducing conditions (**a**) and 10% native-PAGE (**b**). (**c**) Size exclusion chromatography (SEC) analysis of purified OsAPX2. The separated proteins were collected in four fractions (F1–F4) for further analysis. The values in the chromatogram represent the molecular weights of the protein standards: blue dextran (>2000 kDa), thyroglobulin (669 kDa), ferritin (440 kDa), aldolase (158 kDa), ovalbumin (44 kDa) and carbonic anhydrase (29 kDa). Structural analysis of the OsAPX2 protein fractions (inset). Each fraction corresponds to a peak from SEC (F1–F4) and these were analysed using 10% native-PAGE (upper panel) or 12% SDS-PAGE (lower panel).

Figure 1 shows the structural analyses of recombinant OsAPX2 protein using reducing SDS-PAGE (Fig. 1a) and native-PAGE (Fig. 1b) followed by SEC (Fig. 1c). Large amounts of purified protein were obtained under reducing conditions and the OsAPX2 protein was present as a monomer of approximately 27 kDa (Fig. 1a). On the native gel, OsAPX2 protein was present in an LMW form and in HMW complexes (Fig. 1b). To investigate the native structures of these proteins in more detail, we performed SEC analysis using FPLC (Fig. 1c). The OsAPX2 proteins were separated into four fractions corresponding to the major dimeric form (F4), the LMW forms (F2 and F3), and the HMW complexes (F1). Each of these fractions corresponded to a peak in the SEC analysis and was further analyzed by native-PAGE. The proteins in the first SEC fraction (F1 > 2,000 kDa), which contained the largest OsAPX2 complexes, were too large for 10% native-PAGE and were retained at the top of the separating gel (Fig. 1c, inset). The F2 and F3 forms had molecular weights between 158 and 440 kDa. F2 and F3 have a molecular weight close to 440 kDa and 158 kDa, respectively. Although it is difficult to precisely separate F2 and F3, F2 has a molecular weight greater than F3. The major-peak (F4) proteins eluted at a position corresponding to the dimeric forms (approximately 54 kDa). In contrast to the native-PAGE analysis, reducing SDS-PAGE of all fractions resulted in a single 27 kDa band corresponding to the OsAPX2 protein monomer (Fig. 1c, inset, lower panel).

These observations suggest that native OsAPX2 exists as a homo-oligomer consisting of a number of OsAPX2 monomeric units. The structure of OsAPX2 resembles that of the 2-Cys Prx protein, which has dual functions as a peroxidase and molecular chaperone^14–17^. Our results suggest that the structural status of the OsAPX protein may also be important.

### OsAPX2 has dual functions as an APX and molecular chaperone

**Figure 2:**
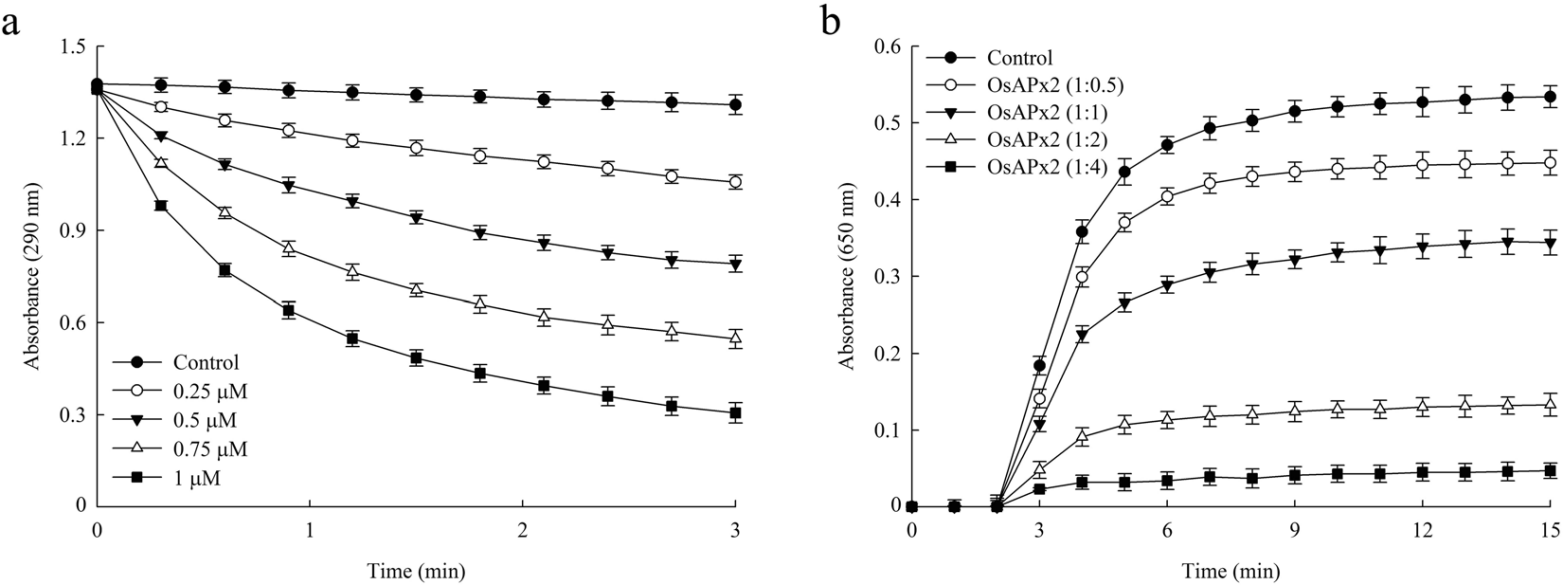
Enzymatic activity analysis of the recombinant OsAPX2 protein. (**a**) The APX activity of purified OsAPX2 protein. The concentrations of purified OsAPX2 used were: 0.25 mM (○), 0.5 µM (▼), 0.75 µM (Δ), 1 µM (■) and 0 µM (●) OsAPX2 protein. (**b**) Molecular chaperone activity of purified OsAPX2 protein. The molecular chaperone activity of OsAPX2 protein was measured using malate dehydrogenase (MDH) as a substrate. Thermal aggregation of MDH was induced at 43 °C for 15 min. The control plot (●) shows the thermal aggregation of MDH in the absence of OsAPX2. The molecular chaperone activity of OsAPX2 was evaluated at 1:0.5 (○), 1:1 (▼), 1:2 (Δ), and 1:4 (■) molar ratios MDH:OsAPX2. The data are expressed as means of at least three independent experiments.

APX enzymes play an important role in the removal of H_2_O_2_ as part of the ascorbate-glutathione or Asada-Halliwell-Foyer pathway^18,19^. We confirmed that the recombinant OsAPX2 protein functions as a typical APX by monitoring the decrease in absorbance at 290 nm caused by ascorbate oxidation^20^. As shown in Fig. 2a, APX activity increased in a dose-dependent manner, demonstrating that OsAPX2 can function as a H_2_O_2_ scavenger.

To investigate whether OsAPX2 can also act as a molecular chaperone, we performed the chaperone activity assay using MDH as a heat-sensitive substrate. OsAPX2 showed strong chaperone activity, and increasing the concentration of OsAPX2 in the assay mixture significantly inhibited aggregation of the MDH substrate protein at 43 °C (Fig. 2b). The aggregation of MDH was effectively suppressed at a 1:4 molar ratio of MDH:OsAPX2 (Fig. 2b). Therefore, our novel finding demonstrates that OsAPX2 functions both as an APX and as a molecular chaperone.

### The functions of OsAPX2 were closely associated with its structural status

To investigate the relationship between the structure and function of OsAPX2, we examined the APX and chaperone activities in each of the F1 to F4 fractions. To test whether the structures of proteins in each fraction were stable, the protein fractions were concentrated and subjected to re-chromatography using the original SEC conditions. We found that the proteins were eluted with nearly the same retention times that they had in the first SEC analysis (Fig. 1c), which suggests that the structures of these proteins remained relatively stable during experimentation *in vitro* (data not shown).

**Figure 3:**
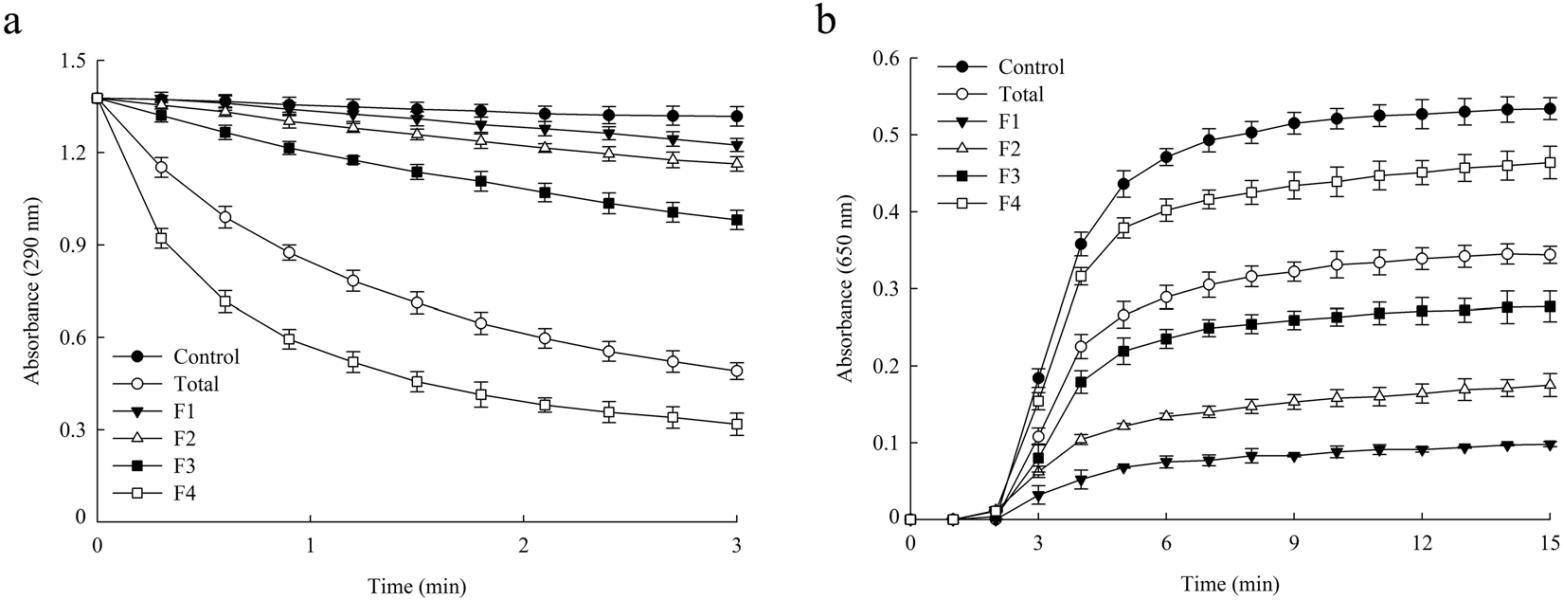
Functional analysis of OsAPX2 fractions. The relative APX (**a**) and molecular chaperone (**b**) activities of the four OsAPX2 protein fractions were compared with those of total protein (○) and no protein (●). The OsAPX2 proteins were separated into four fractions corresponding to the major dimeric form F4 (□), the LMW forms F2 (Δ) and F3 (■), and the HMW complexes F1 (▼). Molecular chaperone activity was evaluated using a 1:1 molar ratio of MDH and OsAPX2. The data are expressed as means of at least three independent experiments.

Figure 3a illustrates that APX activity in the F4 fraction (which consisted mainly of dimeric protein; Fig. 1c) was highest compared with the other fractions and with the total protein extract. Moreover, the APX activity in the other fractions was lower than in the total protein extract (Fig. 3a). In contrast, the chaperone activity was lowest in the F4 fraction (Fig. 3b), whereas the HMW protein complexes in the F1 fraction had higher chaperone but lower APX activity than the total protein extract (Fig. 3). These results suggest that oligomerization of the protein complexes promotes molecular chaperone activity whereas the LMW species are more closely associated with APX activity. Therefore, the dual function of OsAPX2 is linked to its ability to form distinct protein structures.

### The APX activity of OsAPX2 is not required for its function as a molecular chaperone

**Figure 4:**
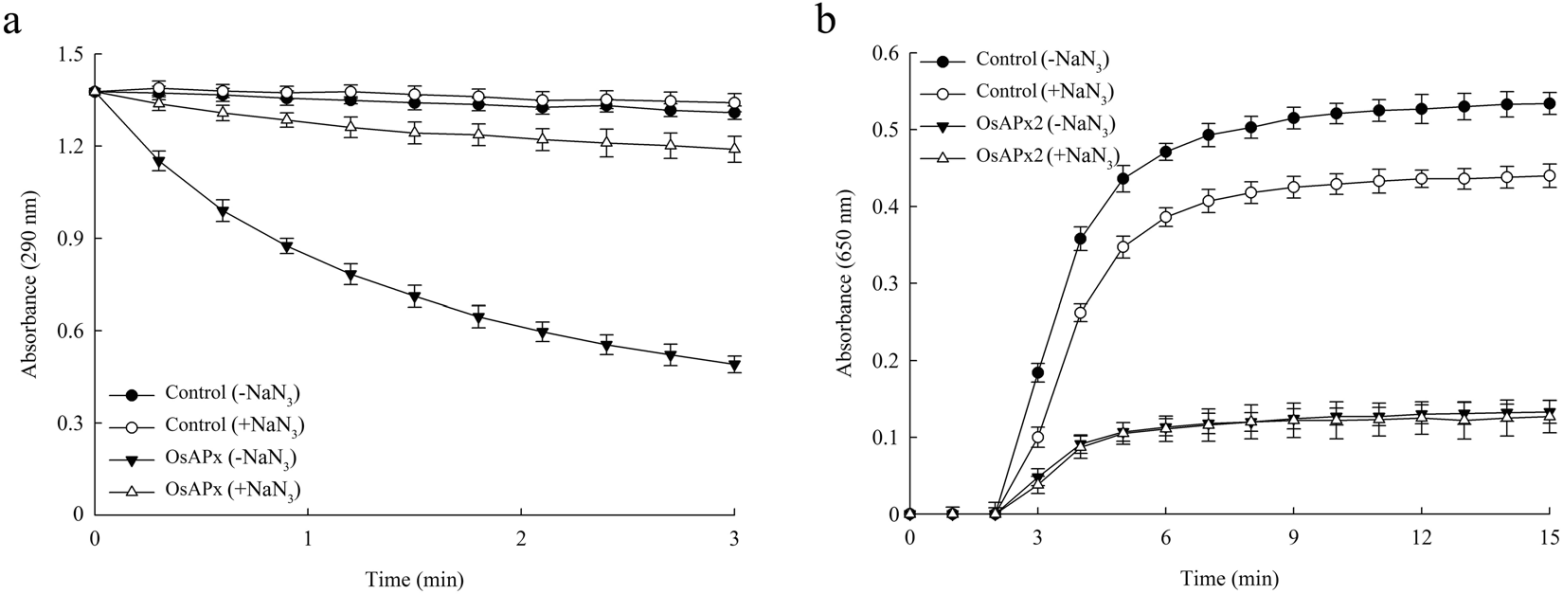
Comparison of APX and molecular chaperone activities in functional OsAPX2 (▼) and haem-inhibited OsAPX2 proteins (Δ). To inhibit haem, we incubated OsAPX2 proteins with 1 mM sodium azide (NaN_3_) for 10 min. Molecular chaperone activity was evaluated using a 1:1 molar ratio of MDH and OsAPX2. The data are expressed as means of at least three independent experiments.

APXs contain the haem moiety ferriprotoporphyrin IX and this plays an important role in their enzymatic activity, which is the breakdown of hydrogen peroxide by the preferential oxidation of ascorbate^20–22^. Sodium azide (NaN_3_) can inhibit a number of enzymes that contain the haem prosthetic group, including peroxidases, superoxide dismutase and catalase. Plant APXs can be inhibited by both azide and cyanide^23,24^. In this study, we identified a new function for OsAPX2 by demonstrating that it can also act as a molecular chaperone. Therefore, to investigate the relationship between its APX and chaperone activities, we assessed the functional capacities of OsAPX2 following 10 min of 1 mM sodium azide treatment. The APX activity was effectively suppressed by NaN_3_ treatment (Fig. 4a). However, the molecular chaperone activity of OsAPX2 treated with NaN_3_ was similar to that of untreated protein (–NaN_3_) (Fig. 4b). Therefore, we suggest that OsAPX2’s function as a molecular chaperone does not depend on its APX activity or the haem group.

### Structural changes in OsAPX2 can be modulated by salt and heat stresses

**Figure 5:**
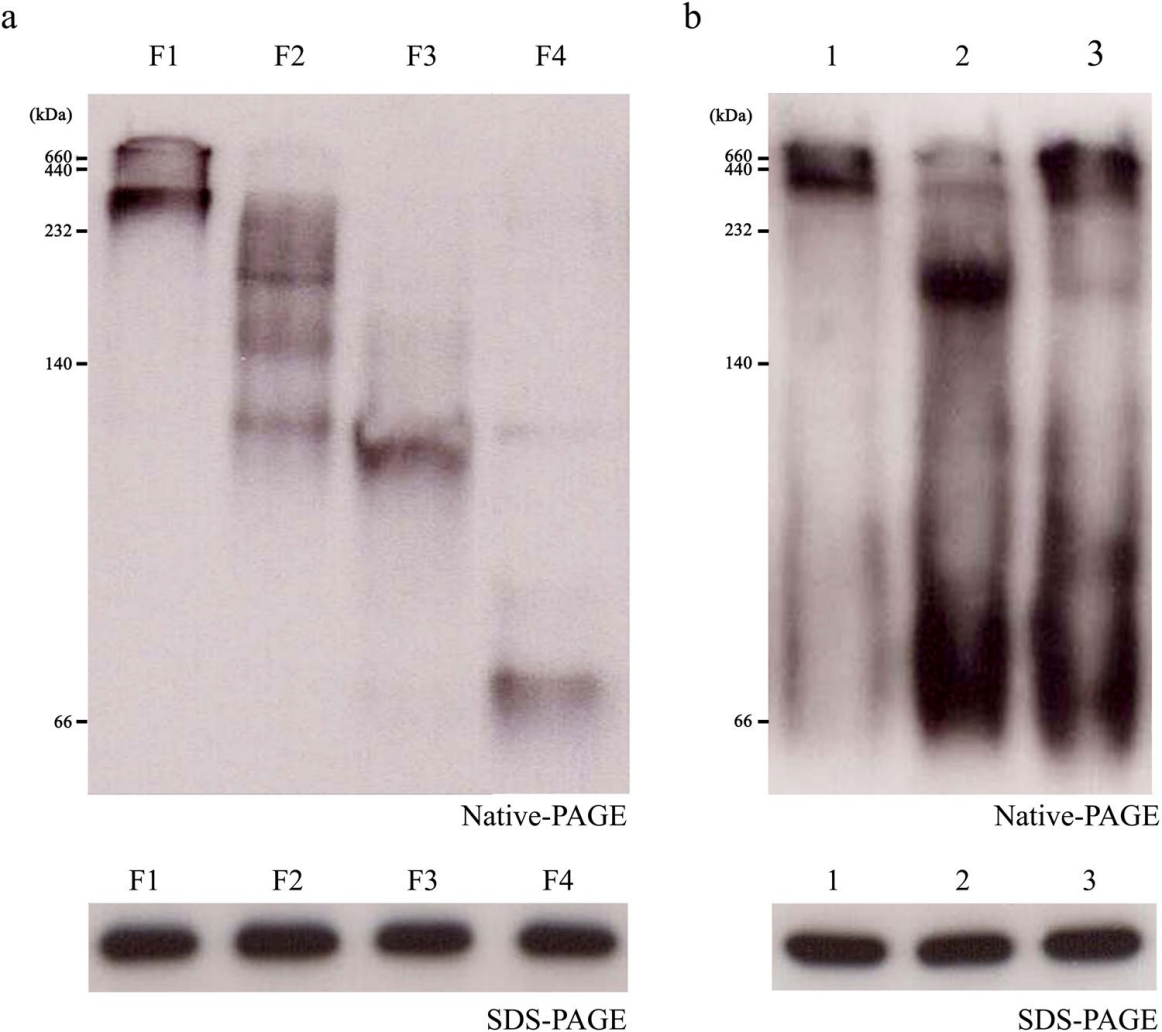
Comparison of OsAPX2 fractions and molecular switching of OsAPX2 in response to salt treatment and recovery. Each fraction was analysed by western blotting with polyclonal anti-OsAPX2 antibodies after separation using 10% native-PAGE (upper panel) or 12% SDS-PAGE under reducing conditions (lower panel). (**a**) Structural analysis of fractionated OsAPX2 proteins by western blotting. The OsAPX2 proteins were separated into four fractions corresponding to the major dimeric form (F4), the LMW forms (F2 and F3), and the HMW complexes (F1). (**b**) Molecular switching of OsAPX2 in response to salt-stress treatment and recovery in IR29. Rice seedlings were cultivated in 0.5× Murashige and Skoog (MS) liquid medium for 3 weeks (Lane 1). For salt stress, 3-week-old cultivated rice plants were transferred to nutrient solution (0.5× MS medium) containing 100 mM NaCl for 3 d (Lane 2). For recovery, rice plants treated with salt stress were transferred to a new nutrient solution containing no salt and grown for 3 d (Lane 3).

In this study, we demonstrated that OsAPX2 can function as both an APX and a molecular chaperone and that these functions are closely linked to the protein’s structural status. Therefore, we investigated factors that might regulate changes in the OsAPX2 structure *in vivo*. Salt-sensitive rice cultivar IR29 plants were grown in a greenhouse for 3 weeks in 0.5× MS nutrient solution and then treated for 3 d with 100 mM NaCl before a final 3 d recovery period in 0.5× MS nutrient solution. Crude protein extracts were subjected to native-PAGE followed by western blotting analysis using a polyclonal anti-OsAPX2 antibody (Fig. 5b). In contrast to the structures of the recombinant OsAPX2 proteins (Fig. 1b,c), these proteins mainly formed HMW complexes with very few LMW proteins present *in vivo* (Fig. 5b). When exposed to salt stress, most of the HMW complexes broke down into the dimeric and LMW forms. In the absence of salt stress, they partly reverted to their original structures. According to Jang *et al*.^15^, oxidative stress and heat shock result in the conversion of yeast 2-Cys Prx from a low molecular weight (LMW) form to high molecular weight (HMW) complexes. Salt treatment could cause oxidative stress. However, salt stress disrupted the majority of the OsAPX2 HMW complexes, which transformed into the dimeric and LMW forms, indicating that the regulation of OsAPX2 activity might differ from that of 2-Cys Prx activity. Similar to OsAPX2, following exposure to H_2_O_2_, *Pseudomonas aeruginosa* 2-Cys Prx is converted from a HMW form to a LMW form. This change triggers a functional switch form chaperone to peroxidase^25^. Although the factors causing the structural changes in yeast 2-Cys Prx and rice OsAPX2 differ, it is widely believed that the LMW form functions as a peroxidase and that the HMW form acts as a chaperone.

We also explored structural alterations in the OsAPX2 protein in response to heat stress. Figure 5b shows that most OsAPX2 proteins form HMW complexes *in vivo*, making it difficult to distinguish structural changes occurring in response to heat stress. Therefore, we investigated changes in the structure of purified OsAPX2 proteins induced by heat stress *in vitro*. The purified OsAPX2 proteins were treated for 10 min at various temperatures. As the temperature was increased, the peak corresponding to HMW complexes dramatically increased and there was a corresponding decrease in the level of LMW proteins (Supplementary Fig. S1a). To demonstrate the stability of heat-treated OsAPX2, we performed native- and SDS-PAGE following heat treatment. OsAPX2 remained stable following heat treatment up to 50 °C (Supplementary Fig. S1b). Additionally, the molecular chaperone activity of OsAPX2 gradually increased following heat treatment (Supplementary Fig. S1d), whereas the APX activity of the protein decreased with increasing temperature (Fig. 1c). These results suggest that the dual functions of OsAPX2 are closely linked to conformational changes in the protein that are modulated by salt (dissociation) and heat (association) stresses.

## Discussion

APX is one of the key enzymes regulating the level of H_2_O_2_ in cells^26^. In plants, the APXs are classified according to their cellular location, with OsAPX2 being found in the cytosol. This study demonstrates that OsAPX2 can act as a molecular chaperone in addition to its primary function as a peroxidase. To the best of our knowledge, this is the first study reporting the dual functions of OsAPX2 as a molecular chaperone and peroxidase. However, previous studies have shown that 2-Cys Prx, another H_2_O_2_ detoxifying enzyme, has dual functions as a molecular chaperone and peroxidase^13,15,27^. The functional switching of 2-Cys Prx was linked to conformational changes in the protein between its LMW and HMW forms^13,27^. Similar structural changes were observed in OsAPX2, and native-PAGE and SEC analyses showed that OsAPX2 could also exist in different conformational states. Therefore, the dual functionality of OsAPX2 could have a structural basis similar to that underlying the different roles played by 2-Cys Prx proteins.

This study has confirmed that the dual functions of OsAPX2 are closely associated with changes in its protein structure. Extracts of the recombinant OsAPX2 protein were separated into different fractions based on molecular weight. The fraction containing predominantly dimeric proteins showed the highest APX activity, whereas the fraction containing mostly HMW complexes had the highest chaperone activity. These results clearly indicate that oligomerization of the OsAPX2 protein promotes molecular chaperone activity, whereas the LMW forms are more strongly linked with APX activity. Therefore, as with the 2-Cys Prx proteins, the dual functions of OsAPX2 are associated with the protein’s capacity to adopt different structural conformations^13^.

APX contains a haem group that plays a key role in H_2_O_2_ detoxification^20^, whereas particular regions of the polypeptide chain may be more important for other functions of OsAPX2, including its role as a molecular chaperone. Sodium azide inhibits haem-containing enzymes^24^, and treating the OsAPX2 protein with sodium azide completely inhibited its APX activity but did not affect its activity as a molecular chaperone. Because the molecular chaperone activity of sodium azide-treated OsAPX2 protein was unaffected, this activity does not require a functional haem group.

The structural changes in the OsAPX2 protein can apparently be modulated by different environmental conditions. We monitored the *in vivo* structural status of OsAPX2 under various abiotic stress conditions and found that it could be altered by salinity stress. OsAPX2 proteins extracted from the salt-sensitive rice cultivar IR29 were mainly present in the HMW form, whereas exposure to salt stress converted them into the dimeric and LMW forms that are associated with APX activity. The proteins reverted to their predominantly HMW form when the salt stress was withdrawn. Lee *et al*.^28^ reported total APX activity, redox status, and H_2_O_2_ content in the salt-sensitive rice seedling IR-29 following salt treatment (100 mM NaCl). Total APX activity was significantly increased in the leaves and roots of IR-29 following salt treatment. The ratio of reduced/oxidized ascorbate was also reduced in IR-29. As ascorbate is oxidized in an H_2_O_2_-scavenging reaction by APX, the decreased ratio is likely due to the increase in APX activity. Additionally, the H_2_O_2_ content of the leaves and roots of IR-29 was decreased. The low H_2_O_2_ content following salt treatment could be partially accounted for by the increased H_2_O_2_-scavenging activity. Therefore, our results can be linked to the functional and structural changes of OsAPX2 in IR-29 following salt stress. Under salt stress, it is likely that the HMW complexes of OsAPX2 can switch to the LMW form to increase APX activity.

In addition, to investigate the MW switching of OsAPX2 in a salt-insensitive rice cultivar in response to salt stress, we selected the salt-tolerant rice cultivar Pokkali, which was obtained from the International Rice Research Institute (Los Baños, Philippines). To determine the structural status of OsAPX2 and its response to salt treatment (100 mM NaCl), we performed native-PAGE and western blot analysis (Supplementary Fig. S2). Like IR-29, under normal condition, the OsAPX2 in the salt-tolerant cultivar Pokkali was present primarily in HMW complexes. In IR-29 (salt-sensitive), OsAPX2 mainly formed HMW complexes with very few LMW proteins; salt stress broke down the majority of the HMW complexes into dimeric and LMW forms. However, unlike in IR-29, the protein structure in Pokkali did not change following salt treatment with 100 mM NaCl and subsequent recovery. Lee *et al*.^28^ reported that two rice cultivars, IR-29 and Pokkali, undergo different morphological and biochemical changes under salt stress. Following salt treatment with 100 mM NaCl, IR-29 showed structural damage to chloroplasts and a decrease in photosynthetic activity but showed little physiological damage in Pokkali despite increased levels of H_2_O_2_. The MW switching of OsAPX2 from HMW complexes to dimeric and LMW forms seems to occur in salt-sensitive rice to overcome salt stress in conjunction with increased APX activity.

**Figure 6:**
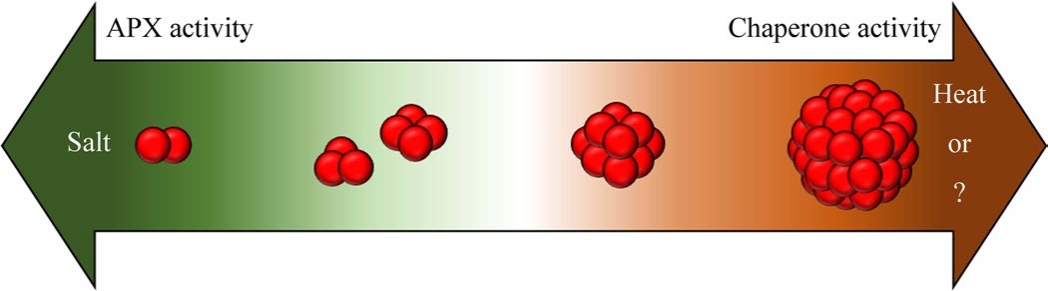
Model showing the salt- and heat-dependent structural changes that occur during the conversions between the LMW and HMW forms of OsAPX2. OsAPX2 is predominantly found as HMW complexes *in vivo*. The model also highlights the functional changes in OsAPX2 that are associated with the structural changes in the protein.

In the present study, because most OsAPX2 proteins present *in vivo* adopted the HMW form, purified OsAPX2 was used to investigate the effect of heat stress. The data showed that increasing the temperature gradually increased the proportion of HMW OsAPX2 and there was a corresponding decrease in the LMW protein form. We can conclude that OsAPX2 has dual functions as both a chaperone and APX and that these are associated with the HMW and LMW forms of the protein, respectively. Additionally, structural changes in the protein can be triggered by external environmental conditions. However, further experiments will be required to describe precisely the mechanisms that modulate the structural and functional alterations in OsAPX2 that are stimulated by changes in temperature and salinity (Fig. 6). In the present study, we demonstrated that the purified OsAPX2 protein exhibited a new function as a molecular chaperone (Fig. 6). Molecular chaperone activity was exhibited predominantly by the HMW form, which was prone to switching by heat stress *in vitro*. However, it was difficult to prove the MW switching of OsAPX2 following heat stress in rice. Since the predominant form of OsAPX2 was the HMW form, a different form induced by heat stress, in normal conditions *in vivo*. We do not currently understand why the mechanism of association of OsAPX2 is different *in vitro* and *in vivo*. It will be necessary to explore this in future studies. However, the present study strongly suggests that OsAPX2 has various structures, that the LMW form functions as a strong peroxidase, and that the HMW form acts as a molecular chaperone (Fig. 6).

## Methods

### Reagents

The *Escherichia coli* strains DH5α (Promega, Madison, WI, USA) and BL21 (DE3) (Invitrogen, Carlsbad, CA, USA) were used in this study. The transformed bacteria were grown in Luria-Bertani medium (DB, Franklin Lakes, NJ, USA) and isopropyl-1-thio-β-D-galactopyranoside (IPTG) was purchased from Promega. Ascorbic acid, bovine serum albumin (BSA), ethylenediaminetetraacetic acid (EDTA), hydrogen peroxide (H_2_O_2_), nicotinamide adenine dinucleotide phosphate (NADPH), phenylmethylsulfonyl fluoride (PMSF) and skimmed milk were all purchased from Sigma-Aldrich (St. Louis, MO, USA), and the protein molecular size standards used for PAGE were obtained from ELPIS (Daejeon, South Korea). Bradford protein assay solution was purchased from Bio-Rad (Hercules, CA, USA) and malate dehydrogenase (MDH) was prepared as described previously^29^.

### Cloning the *OsAPX2* gene and protein expression in *E*. *coli*

The *OsAPX2* gene from *Oryza sativa* was cloned into the pGEM-T easy vector (Promega) and subsequently subcloned into the pET28a vector (Novagen, Madison, WI, USA). The *OsAPX2*-containing pET28a plasmid was then transformed into *E*. *coli* BL21 (DE3) and overexpressed. His-tagged OsAPX2 protein was purified using a metal-chelate affinity chromatography system with nickel-nitrilotriacetate agarose (Peptron, Daejeon, South Korea) and phosphate-buffered saline containing 1 mM ascorbate. The purified protein was dialysed into 50 mM Tris-HCl buffer (pH 7.5), and the protein concentration was determined using the Bradford dye-binding method with BSA protein standards^30^.

### APX activity assay

APX activity was determined using ascorbate oxidation according to the method described previously^20^. Enzyme activity was monitored by measuring the decrease in absorbance at 290 nm for 3 min immediately after adding 1 mM H_2_O_2_ to a 500 µL reaction mixture containing 0.25 mM ascorbate, 100 mM potassium phosphate buffer (pH 7.0) and OsAPX2 protein. Sodium azide (1 mM NaN_3_) was used as an inhibitor of the haem-containing APX protein.

### Molecular chaperone activity assay

Molecular chaperone activity was quantified by determining the prevention of thermal aggregation of a heat-sensitive substrate protein (MDH). The MDH was incubated with various concentrations of OsAPX2 recombinant protein in 50 mM HEPES at 43 °C. The thermal aggregation of the substrate was monitored for 15 min at an absorbance of 650 nm using an Evolution 300 UV-Vis spectrophotometer equipped with a thermostatic cell holder (Thermo Scientific, Worcester, MA, USA).

### SEC

Determination of the molecular weight and purification of the OsAPX2 protein was performed by SEC using a Superdex 200 10/300 GL gel-filtration column (Amersham Biosciences, Uppsala, Sweden) and an AKTA fast protein liquid chromatography (FPLC) system (Amersham Biosciences). The column was pre-equilibrated with 50 mM Tris-HCl buffer (pH 7.5) containing 100 mM NaCl and the flow rate was 0.5 mL/min at 4 °C. The absorbance was monitored at 280 nm. Fractions containing the desired protein (F1–F4) were pooled, concentrated and analysed. The column was calibrated using blue dextran (>2,000 kDa), thyroglobulin (669 kDa), ferritin (440 kDa), aldolase (158 kDa), conalbumin (75 kDa), ovalbumin (44 kDa) and carbonic anhydrase (29 kDa).

### Plant materials and growth conditions

IR29 (salt-sensitive rice cultivar) and Pokkali (salt-tolerant rice cultivar) were obtained from the International Rice Research Institute (Los Baños, Republic of the Philippines). Rice seeds were surface-sterilized using 2.5% sodium hypochlorite solution and rinsed in distilled water. Seeds were germinated in distilled water at 30 °C for 3 d under dark conditions. The seedlings were then transferred to holes in styrofoam boards lined with a nylon net and floating on a nutrient solution containing 0.5× MS medium. They were grown for 3 weeks in a 12 h photoperiod (30 °C day/25 °C night, 700–800 µmol m^−2^ S^−2^ light intensity, and 70–80% humidity). The nutrient solution was replaced every week.

### Salt and heat stress treatments and recovery

To induce salt stress, 3-week-old rice seedlings were transferred to a nutrient solution (0.5× MS medium) containing 100 mM NaCl for 3 d. After the salinity treatment, the rice seedlings were separated into two groups. One group of seedlings was processed for further analysis and the other was transferred to fresh nutrient solution with no salt and allowed to recover for 3 d. The nutrient solution for the recovering plants was replaced every 2 d, and all experiments were repeated three times. To induce heat stress, the purified OsAPX2 protein was exposed to 25 °C (untreated), 30 °C, 40 °C, and 50 °C for 10 min. all experiments were repeated at least three times.

### Total plant protein extracts for western blotting

IR29 shoot tissue (0.3 g fresh weight per sample) was immediately frozen upon harvesting and ground to a fine powder under liquid nitrogen. A total of 0.5 mL of protein extraction buffer (50 mM Tris-HCl pH 8.0, 0.25 M sucrose, 2 mM EDTA, and 1 mM PMSF) was added to each sample and incubated at 4 °C for 2 h. The homogenate was centrifuged at 15,000 g for 20 min and the supernatant was retained for analysis. The protein content of this total protein extract was measured using the Bradford dye-binding method with BSA protein standards^26^. To investigate switching between the different MW OsAPX2 complexes, we performed western blot analysis using both reducing 12% sodium dodecyl sulphate (SDS)-PAGE and 10% native-PAGE. The western blot membranes with bound total protein extracts were probed using polyclonal OsAPX2 antibodies. Reducing SDS-PAGE and native-PAGE were performed as described previously^31^.

## Electronic supplementary material

Supplementary information
