# Supplementary material for: Functional switching of ascorbate peroxidase 2 of rice (OsAPX2) between peroxidase and molecular chaperone
Source: Sci Rep. 2018 Jun 15;8:9171. doi: 10.1038/s41598-018-27459-1 (PMC6003922; doi:10.1038/s41598-018-27459-1)
Supplement: Supplementary file 1 — Supplementary information [file 41598_2018_27459_MOESM1_ESM.pdf]

Supplementary information

Functional switching of ascorbate peroxidase 2 of rice (OsAPX2) between peroxidase and molecular chaperone

Sung Hyun Hong[1], Bhumi Nath Tripathi[1], Moon-Soo Chung[1], Chuloh Cho[1], Sungbeom Lee[1,4], Jin-Hong Kim[1,4], Hyoung-Woo Bai[1,4], Hyeun-Jong Bae[2], Jae-Young Cho[3], Byung Yeoup Chung[1] & Seung Sik Lee[1,4,*]

[1]Advanced Radiation Technology Institute, Korea Atomic Energy Research Institute, 29 Geumgu-gil, Jeongeup 580-185, Korea. [2]Department of Bioenergy Science and Technology, Chonnam National University, Gwangju 61186, Korea. [3]Department of Bioenvironmental Chemistry, Chonbuk National University, 567 Baekje-daero, Deokjin-gu, Jeonju 54896, Korea. [4]Department of Radiation Biotechnology and Applied Radioisotope, Korea University of Science and Technology, Daejeon 34113, Korea. *Correspondence and requests for materials should be addressed to S.S.L. (email: sslee@kaeri.re.kr)

Supplementary Figure S1

Supplementary Figure S2

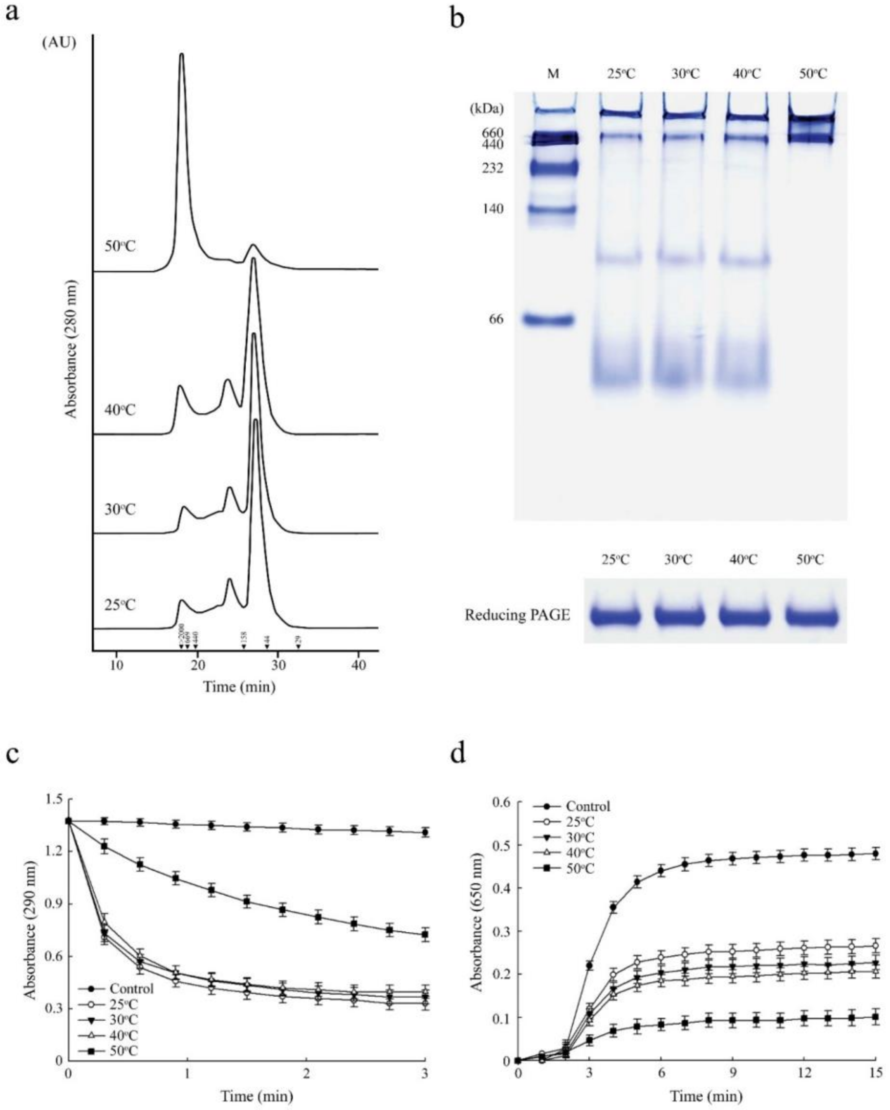

Supplementary Figure S1

Structural and functional changes of purified OsAPX2 protein in response to heat treatment. Structural analysis of heat-treated OsAPX2 protein using SEC (a) and PAGE (b). The heat-treated OsAPX2 proteins were analysed using 10% native-PAGE (upper panel) or 12% SDS-PAGE (lower panel) (b). The relative APX (c) and molecular chaperone (d) activities of heat-treated OsAPX2 proteins were compared with those of untreated (25℃) (○) proteins. Purified OsAPX2 protein was incubated for 10 min at 30℃ (▼), 40°C (Δ) or 50°C (■). The data are expressed as means of at least three independent experiments.

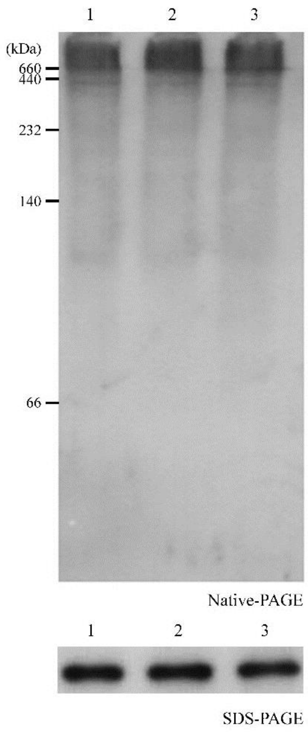

Supplementary Figure S2

Supplementary Fig. S2. Molecular switching of OsAPX2 in response to salt-stress treatment and recovery in salt-insensitive rice cultivar (Pokkali). Rice seedlings were cultivated in 0.5× MS liquid medium for 3 weeks (Lane 1). For salt stress, 3-week-old cultivated rice plants were transferred to nutrient solution (0.5× MS medium) containing 100 mM NaCl for 3 d (Lane 2). For recovery, rice plants treated with salt stress were transferred to a new nutrient solution containing no salt and grown for 3 d (Lane 3).
